# Enhancement of Thiamine Biosynthesis in Oil Palm Seedlings by Colonization of Endophytic Fungus *Hendersonia toruloidea*

**DOI:** 10.3389/fpls.2017.01799

**Published:** 2017-10-17

**Authors:** Amirah N. Kamarudin, Kok S. Lai, Dhilia U. Lamasudin, Abu S. Idris, Zetty N. Balia Yusof

**Affiliations:** ^1^Department of Biochemistry, Faculty of Biotechnology and Biomolecular Sciences, Universiti Putra Malaysia, Serdang, Malaysia; ^2^Ganoderma and Diseases Research Group, Biology Division, Malaysian Palm Oil Board, Kajang, Malaysia; ^3^Department of Cell and Molecular Biology, Faculty of Biotechnology and Biomolecular Sciences, Universiti Putra Malaysia, Serdang, Malaysia

**Keywords:** oil palm, endophytic fungi, thiamine biosynthesis, gene expression, endophytic colonization

## Abstract

Thiamine, or vitamin B1 plays an indispensable role as a cofactor in crucial metabolic reactions including glycolysis, pentose phosphate pathway and the tricarboxylic acid cycle in all living organisms. Thiamine has been shown to play a role in plant adaptation toward biotic and abiotic stresses. The modulation of thiamine biosynthetic genes in oil palm seedlings was evaluated in response to root colonization by endophytic *Hendersonia toruloidea*. Seven-month-old oil palm seedlings were inoculated with *H. toruloidea* and microscopic analyses were performed to visualize the localization of endophytic *H. toruloidea* in oil palm roots. Transmission electron microscopy confirmed that *H. toruloidea* colonized cortical cells. The expression of thiamine biosynthetic genes and accumulation of total thiamine in oil palm seedlings were also evaluated. Quantitative real-time PCR was performed to measure transcript abundances of four key thiamine biosynthesis genes (*THI4*, *THIC*, *TH1*, and *TPK*) on days 1, 7, 15, and 30 in response to *H. toruloidea* colonization. The results showed an increase of up to 12-fold in the expression of all gene transcripts on day 1 post-inoculation. On days 7, 15, and 30 post-inoculation, the relative expression levels of these genes were shown to be downregulated. Thiamine accumulation was observed on day 7 post-colonization and subsequently decreased until day 30. This work provides the first evidence for the enhancement of thiamine biosynthesis by endophytic colonization in oil palm seedlings.

## Introduction

**FIGURE 1:**
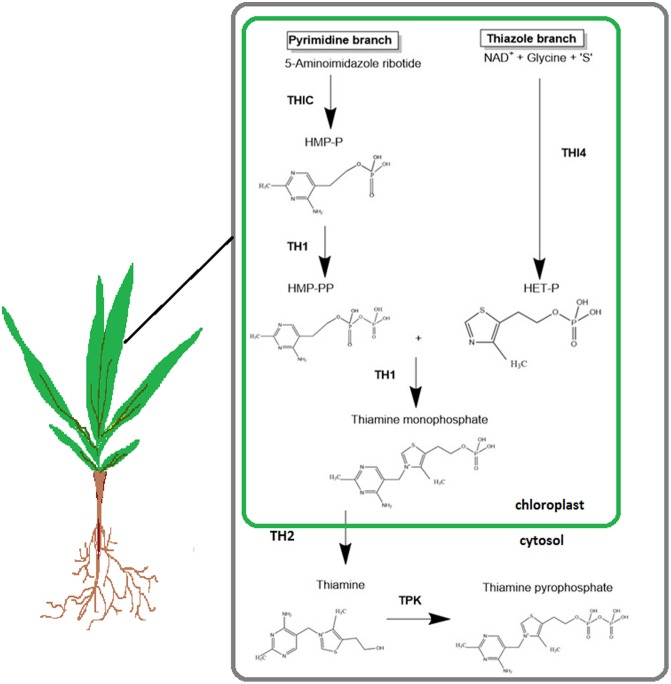
The thiamine biosynthesis pathway in plants. HMP-P, hydroxymethylpyrimidine phosphate; HMP-PP, hydroxymethylpyrimidine pyrophosphate; HET-P, hydroxyethylthiazole phosphate; THIC, hydroxymethylpyrimidine synthase; THI4, thiazole biosynthetic protein; TH1, hydroxymethylpyrimidine phosphate kinase; TH2, thiamine monophosphate phosphatase; TPK, thiamine pyrophosphate kinase. [Adapted from Kamarudin et al. (2017). The figure is reproduced with permission from the copyright holder.]

Thiamine, also known as vitamin B1, is required for key metabolic processes in cellular organisms. The active form, thiamine pyrophosphate (TPP), is a cofactor in important metabolic reactions, notably glycolysis, tricarboxylic acid cycle, pentose phosphate pathway, and synthesis of branched amino acids (Goyer, 2010). The thiamine biosynthesis pathway in plants is similar to that in bacteria and yeast (Begley et al., 1999; Li et al., 2010). As shown in **Figure 1**, this pathway consists of two separate branches: the thiazole branch and pyrimidine branch. The pyrimidine moiety of thiamine, hydroxymethylpyrimidine phosphate (HMP), is produced from the precursor 5-aminoimidazole ribonucleotide (AIR) by the enzyme HMP synthase, encoded by the *THIC* gene. The thiazole moiety arises from NAD, glycine, and an S-donor, which form hydroxyethylthiazole phosphate; this is synthesized by the enzyme hydroxyethylphosphate synthase (THI4). The thiazole and pyrimidine moieties are joined together by the bifunctional enzyme (TH1) to form thiamine monophosphate (TMP) (Guan et al., 2014). TMP is dephosphorylated by a phosphatase known as thiamine monophosphate phosphatase (TH2) to form thiamine (Mimura et al., 2016). All of these steps occurs in the chloroplast (Pourcel et al., 2013). The last step is the phosphorylation of thiamine to its active form, TPP by the enzyme thiamine pyrophosphokinase (TPK), which occurs in the cytosol.

Thiamine metabolism is involved in adaptation to biotic and abiotic stresses in plants and microorganisms (Rapala-Kozik et al., 2012; Sylvander et al., 2013). For example, thiamine treatment enhances the resistance of soybean to charcoal rot disease (Monaim, 2011), of rice to sheath blight disease (Bahuguna et al., 2012), and of grapevine to *Plasmapara viticola* (Boubakri et al., 2012). The mechanism of disease suppression through application of thiamine is explained by the activation of a plethora of host defense responses. In *Arabidopsis thaliana*, thiamine treatment activates pathogenesis-related protein (PR-1) and phenylalanine lyase (PAL). In addition, thiamine treatment in grapevine reduces downy mildew development in a dose-dependent manner by inducing hydrogen peroxide generation, callose disposition, and host resistance (HR) cell death. Similarly, thiamine treatment successfully controls charcoal rot disease in soybean plants by inducing defense-related enzymes including peroxidase (PO), polyphenol oxidase (PPO), PAL, and pathogenesis related (PR) chitinase (Monaim, 2011). Therefore, thiamine is thought to be involved in priming, which is important in mitigating crop-damaging diseases and stresses.

Oil palm is the most profitable oil-bearing crop in the world, yielding about 4–6 tons of oil per hectare (Murphy, 2014). However, the productivity of the oil palm is threatened by basal stem rot disease caused by *Ganoderma boninense*, which results in major economic losses and yield gaps (Barcelos et al., 2015). Therefore, studies on the proper management of the disease have been increasing (Hushiarian et al., 2013).

Endophytes, defined as fungi that are present in most plant tissues without causing any visible symptoms, have been utilized as biological control agents in preventive measures against the disease (Wilson, 1995). Several endophytic species have been thus utilized, namely *Actinomycetes*, *Pseudomonas*, *Trichoderma*, and *Hendersonia* (Sapak et al., 2008; Idris et al., 2010; Sundram et al., 2011). It was reported that the application of endophytes results in growth-promoting effects independent of the suppression of *G. boninense*. The endophytic fungus *Hendersonia toruloidea*, originally isolated from oil palm trunk and root tissues, has been used as a biocontrol agent. *In vitro* and nursery trial studies of endophytic application of *H. toruloidea* suppressed infection of the pathogenic fungus *G. boninense* (Idris et al., 2010).

In this study, we examined the responses of oil palm seedlings to colonization by *H. toruloidea*, specifically in terms of the expression of thiamine biosynthetic genes. The morphology and the colonization pattern of *H. toruloidea* were visualized with transmission electron microscopy (TEM). Furthermore, expression of thiamine biosynthetic genes, as well as accumulation of total thiamine and its intermediates, were compared.

## Materials and Methods

### Fungal Strains, Growth Conditions, and Granular Bioformulation Preparation

A strain of the fungal endophyte *H. toruloidea* was previously isolated from healthy oil palms in disease-affected areas of MPOB Teluk Intan, Perak, Malaysia (Idris et al., 2010). Pure axenic cultures were sub-cultured on potato dextrose agar and incubated at 28°C for 10 days. Conidial spores were scraped and poured into potato dextrose broth containing 9% jaggery. Fungal cultures were incubated at 28°C for 4 days in a shaking incubator at 150 rpm. Fungal cultures were encapsulated in an alginate formulation containing kaolin, empty fruit bunch, and pectin (Idris et al., 2010).

### Plant Experimental Conditions

Seven-month-old seedlings of Dura × Pisifera variety oil palms were grown under nursery conditions at MPOB Nursery, Section 15, Bandar Baru Bangi, Selangor, Malaysia. Oil palms were inoculated with *H. toruloidea* by applying 50 g of the bioformulation (10^7^ CFU/g) and drenching with tap water. Control plants were not treated. Spear leaves and roots were sampled (two replicates per treatment in three independent experiments) on days 0, 1, 7, 15, and 30 post-treatment, immediately frozen in liquid nitrogen, and stored at -80°C until further analysis.

### TEM Analysis

Oil palm root sections were cut into 1-mm^3^ slices. Root sections were put into separate vials and fixed in 4% glutaraldehyde for 2 days. Next, the root sections were washed with 0.1 M sodium cacodylate buffer three times for 30 min each. They were then post-fixed in 1% osmium tetroxide for 2 h at 4°C before being washed again with 0.1 M sodium cacodylate buffer three times for 30 min each. A dehydration series of acetone (35, 50, 75, 95%) was used for 45 min each. The final dehydration with 100% acetone was performed three times for 1 h each. Each specimen was then embedded into a beam capsule, which was filled with resin mixture. This was polymerized in an oven at 60°C for 48 h. Thick sectioning was performed by cutting the polymerized specimen into 1-μm thick sections using an ultramicrotome. The thick sections were stained with toluidine blue and dried on a hot plate. The stain was washed under running tap water. The area of interest was examined under a light microscope. For ultrathin sectioning, ultrathin sections were cut and selected for silver staining. The sections were selected with a grid and dried using filter paper. For the staining procedure, the sections were stained with uranyl acetate for 15 min and washed with double-distilled water. Next, they were stained with lead stain for 10 min and washed with double-distilled water. Lastly, the sections were viewed using TEM (Technai G2 Transmission Electron Microscope).

### RNA Isolation and Quantitative Real-time PCR Analysis

Total RNA was isolated from oil palm spear leaves using the CTAB method with modifications (Zeng and Yang, 2002). Genomic DNA was removed using DNase I (Promega, Madison, WI, United States) according to the manufacturer’s instructions. Purified RNA samples (1 μg) were reverse-transcribed using GoScript cDNA synthesis kit (Promega). Specific primers (Supplementary Table S1) for quantitative real-time PCR were designed by Primer Premier 6.0 (Primer Biosoft, Palo Alto, CA, United States). Each 10-μl PCR reaction mixture contained 4.0 μl cDNA template, 5 μl 2× SYBR SensiFast Hi-Rox (Bioline, Taunton, MA, United States), and 0.4 μl 10 mM forward and reverse primers for each gene. Quantitative PCR was performed using a Bio-Rad CFX Connect 96 (Hercules, CA, United States). The cycling conditions were as follows: 2 min at 95°C, followed by 40 cycles of 10 s at 95°C and 30 s at 60°C. Three biological replicates with three technical replicates each were assayed for each sample. Transcript levels of each gene were normalized to the reference genes tubulin and glyceraldehyde-3-phosphate using the method by Vandesompele et al. (2002). The 2^-ΔΔCT^ method was used to analyze the relative changes in expression of *THI4*, *THIC*, *TPK*, and *TH1* (Livak and Schmittgen, 2001).

### Analysis of Thiamine and Its Ester Phosphates by High Performance Liquid Chromatography (HPLC)

For HPLC, 5 g of leaf samples were ground in liquid nitrogen; 20 ml of 0.1 N hydrochloric acid (HCl) was then added and incubated at 37°C for 16 h. Samples were then centrifuged at 4000 × *g* for 10 min. Samples were filtered using Whatman filter paper. Next, 2.5 ml of 5% trichloroacetic acid was added to the filtrate. The derivatization step was performed by adding 2.5 ml of freshly prepared 1% potassium ferricyanide in 15% NaOH, 250 μl of phosphoric acid, and 750 μl of 0.1 N HCl. Samples were filtered using a 0.22-μm syringe filter before being added to amber vials. After sample derivatization into thiochrome and its esters, samples were analyzed by HPLC with fluorescence detection (Agilent 1290 Infinity UPLC, Palo Alto, CA, United States). The column used was a Kinetex 5 μm C18 100 Å, LC column, 100 mm × 4.6 mm (Phenomenex, Torrance, CA, United States). A gradient elution was used, where solvent A contained 10 mM sodium phosphate buffer, pH 7 and solvent B consisted of 100% methanol.

## Results

### Colonization of Oil Palm Seedlings by Endophytic *H. toruloidea*

**FIGURE 2:**
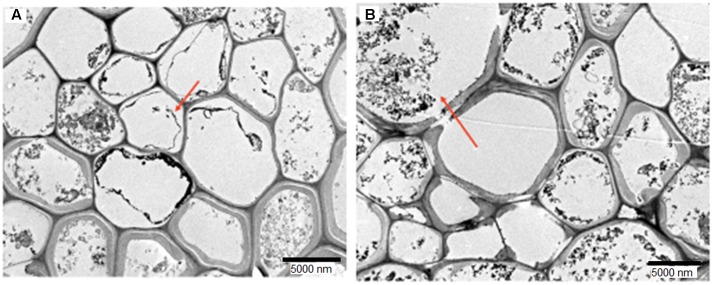
Transverse sections of oil palm root colonized by *Hendersonia toruloidea* at 30 days post-inoculation. Arrow shows hyphal coils **(A)** and spores **(B)** in cortical cells at day 30 of colonization. Bar size is 5000 nm.

The colonization and localization of *H. toruloidea* inside the root was visualized with TEM. **Figure 2** shows a transverse section of oil palm root on days 1 and 30 post-colonization. *H. toruloidea* was found to reside primarily in the cells of the cortical tissues.

### The Effect of *H. toruloidea* Colonization on the Expression of Thiamine Biosynthetic Genes in Oil Palm

**FIGURE 3:**
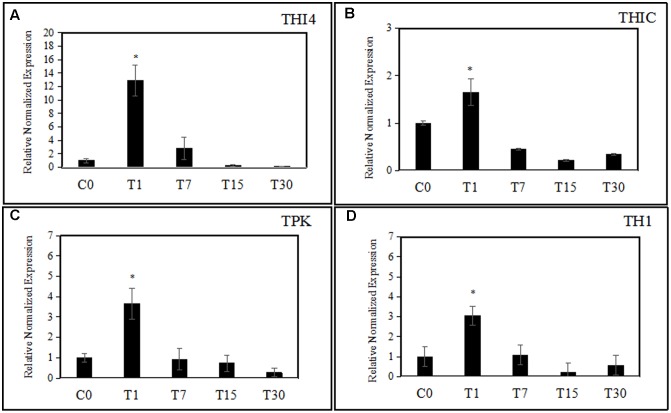
**(A–D)** The effect of colonization of *H. toruloidea* on the expression of *THI4*, *THIC, TH1*, and *TPK*. C0: control, T1: 1 day post-inoculation, T7: 7 days post-inoculation, T15: 15 days post-inoculation, T30: 30 days post-inoculation. Error bars indicated SD of three biological replicates. Asterisks indicate significant differences when compared to controls using Student’s *t*-test (*p* < 0.05).

The expression of thiamine biosynthetic genes in oil palm seedlings during colonization by endophytic *H. toruloidea* was analyzed using quantitative real-time PCR at various time points: 1, 7, 15, and 30 days post-treatment (**Figure 3**). One day after the application of *H. toruloidea*, oil palm seedlings showed 12.9-, 3.65-, 1.65-, and 3.05-fold increased levels of expression of *THI4*, *TPK*, *THIC*, and *TH1*, respectively. Meanwhile on day 7, *THI4*, *TPK*, *THIC*, and *TH1* were downregulated to levels that represented 2.85-, 0.92-, 0.44-, and 1.07-fold changes, respectively, when compared to levels in control seedlings. After 14 days, expression of *THI4*, *THIC*, *TPK*, and *TH1* had continued to decrease to levels that were 0.23-, 0.21-, 0.73-, and 0.21-fold, respectively, those in control seedlings. Finally, at 30 days post-colonization, levels of expression of *THI4*, *THIC*, *TPK*, and *TH1* were even lower, at 0.19-, 0.34-, 0.27-, and 0.57-fold, respectively, those in control seedlings.

### The Effect of *H. toruloidea* Colonization on Total Thiamine Accumulation in Oil Palm Seedlings

**FIGURE 4:**
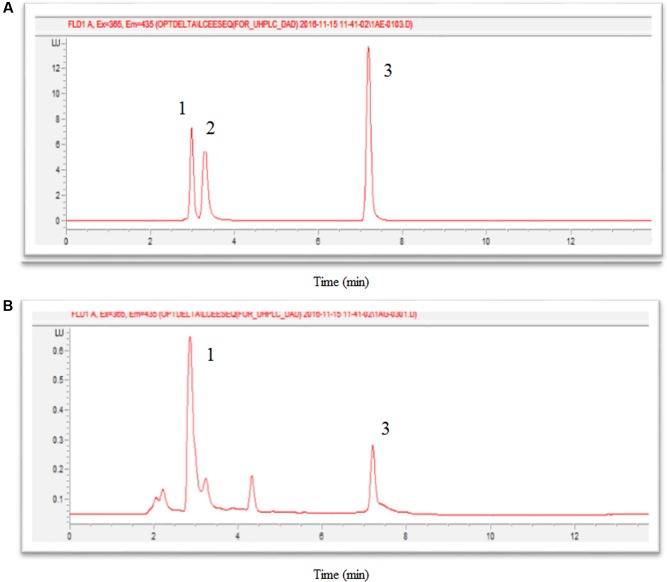
Chromatograms of thiochrome derivatives of thiamine standards **(A)** and oil palm leaves sample **(B)**, where 1 is TPP (Rt = 3.04), 2 is TMP (Rt = 3.35), and 3 is TF (Rt = 7.22).

Well-separated peaks of thiamine (TF), thiamine monophosphate (TMP) and thiamine pyrophosphate (TPP) were detected at retention time (Rt) of 7.22, 3.35, and 3.04 min respectively (**Figure 4A**). **Figure 4B** showed that TMP could not be detected in oil palm leaves.

**FIGURE 5:**
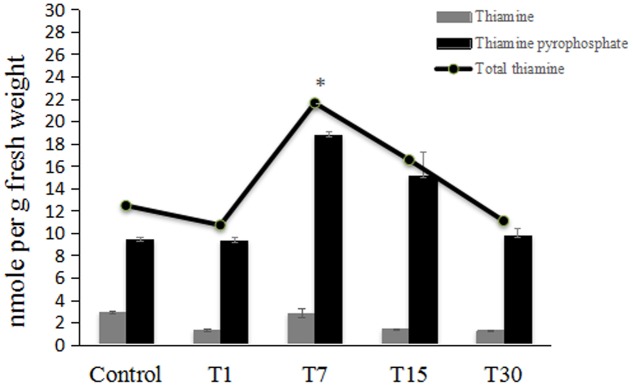
HPLC analysis of thiamine and its intermediates content in control 7-month-old oil palm seedlings (Control) and those inoculated with *H. toruloidea;* T1: 1 day post-inoculation, T7: 7 days post-inoculation; T15: 15 days post-inoculation; and T30: 30 days post-inoculation. In each analysis, 5 g of fresh oil palm leaf was used. Data are means ± SD of three replicates. Asterisks denote significant differences between control and inoculated seedlings (*p* < 0.05).

**Figure 5** summarizes the changes in the contents of total thiamine and its ester phosphates at each time point. The total content of thiamine is the sum of TPP and free thiamine (TF). TPP was present at higher levels than TF. There was no significant increase in total thiamine 1 day post-colonization. Interestingly, the total thiamine content in oil palm leaves was significantly enhanced by twofold on day 7 post-colonization. At subsequent time points (days 15 and 30), the total thiamine content had returned to control levels.

## Discussion

This study was based on the hypothesis that thiamine biosynthesis would be upregulated upon endophytic colonization. Successful colonization by *H. toruloidea* was observed by TEM analysis, which revealed that the fungus resides inside cortical cells. Overall, *THI4*, *TPK*, *THIC*, and *TH1* were upregulated 24 h after inoculation. A previous study had shown that thiamine and its intermediates are involved in systemic acquired resistance in various plant species, acting as signaling molecules (Rapala-Kozik et al., 2008; Tunc-Ozdemir et al., 2009). The observation that thiamine biosynthesis is upregulated as a result of colonization by an endophytic fungus is relatively novel. Because of the relatively long duration of the observation, the downregulation of thiamine biosynthesis 7 days after inoculation can be seen as an adaptation to the establishment of *H. toruloidea* in oil palm seedlings.

We observed that the expression level of *THI4*, which is the first enzyme in the thiamine biosynthesis pathway, was remarkably high 1 day after endophytic colonization by *H. toruloidea*, with an increase of about 12-fold compared to levels of *THIC*, *TPK*, and *TH1*. It is implied that the upregulation of thiamine biosynthetic genes, specifically the increase in *THI4* expression, was caused by the increased demand of TPP, which acts as a coenzyme in numerous physiological processes, including glycolysis, the pentose phosphate pathway, the synthesis of nucleic acids, and the synthesis of NADPH. The increased requirement for TPP-dependent enzymes was probably due to perturbations in metabolism as a result of stress and adaptive responses to *H. toruloidea* colonization in the oil palm seedlings (Rapala-Kozik et al., 2008). Aside from its role as a cofactor, the thiazole synthase family is also involved in other non-cofactor cellular functions. In the yeast *Saccharomyces cerevisiae, THI4* mutants showed higher susceptibility to DNA damage (Machado et al., 1997). Interestingly, it was found that *THI4*, which codes for thiazole synthase in the fungal pathogen *Verticillium dahlia*, is required for stress tolerance against UV damage and vascular disease induction in tomato (Hoppenau et al., 2014). Thus, prominent increase in the expression of *THI4* in response to *H. toruloidea* colonization can be explained by its dual role in thiamine biosynthesis and the stress response.

The application of endophytes is an excellent strategy for activating systemic acquired resistance in plants. Numerous studies have highlighted the potential role of fungal endophytes in plant protection through the upregulation of secondary metabolites, such as phytohormones, phenolic compounds, and defense enzymes (Gao et al., 2010; Kumara et al., 2014). Endophyte-mediated systemic acquired resistance could serve as a disease control strategy in the development of plants with enhanced disease resistance. The relationship between thiamine and systemic acquired resistance was established by Ahn et al. (2007), who showed that thiamine treatment elicited transient expression of PR genes and hypersensitive responses in rice, *Arabidopsis*, and cucumber. Similarly, thiamine treatment in rice induces priming, which results in higher hydrogen peroxide content, total phenolic accumulation, and phenylalanine lyase activity (Bahuguna et al., 2012). Moreover, when the pathogenic fungus *Sclerotinia* is inoculated on *Arabidopsis*, the plant experiences an upregulation in thiamine biosynthesis correlated with increased accumulation of thiamine, TMP, and TPP (Zhou et al., 2013). This implies that there is an increase in the *de novo* synthesis of endogenous thiamine upon *Sclerotinia* infection. Thiamine-treated plants exhibit resistance to the pathogenic fungus by producing reactive oxidative species (ROS) to promote defense signaling. Therefore, in relation to our study, it can be safely assumed that changes in thiamine biosynthesis, specifically the upregulation of *THI4*, are due to early adaptive defense responses in oil palm seedlings upon active colonization by *H. toruloidea*.

Ideally, it is minimally required that both *THIC* and *THI4*, the upstream enzymes for each of the two branches of thiamine biosynthesis (pyrimidine and thiazole), are upregulated in order to increase the total thiamine pool (Pourcel et al., 2013; Dong et al., 2016). We found that *THI4* was significantly upregulated to 12.9-fold after 24 h of endophytic colonization. However, the magnitude of the change in expression of *THIC* upon colonization with the endophytic fungus was not great, at about 1.65-fold. A small increase in the expression of *THIC* compared with that of *THI4* was also demonstrated in oil palm seedlings infected with *G. boninense* (Balia Yusof et al., 2015). This can be explained by the fact that *THIC* is a highly complex energy-expensive enzyme and exhibits a bottleneck in overexpression. The synthesis of *THIC* requires a large energy investment, as structural studies have shown that *THIC* is made of an iron-sulfur cluster, *S*-adenosyl methionine, and ferredoxin–thioredoxin redox system (Colinas and Fitzpatrick, 2015). THIC may also be involved in other functions, such as oxygenic photosynthesis and circadian regulation (Colinas and Fitzpatrick, 2015). Apart from these, THIC, which is responsible for the synthesis of HMP-pyrophosphate (HMP-PP), is required for the synthesis of TMP by condensation of HMP-PP and HET-P. The imbalance in the magnitudes of the expression changes of *THIC* and *THI4* could therefore be explained by the involvement of other sources of HMP-PP. In the yeast (*S. cerevisiae*) thiamine biosynthesis pathway, HMP-PP is obtained from the pyridoxal 5-phosphate (PLP) biosynthesis pathway (Li et al., 2010). This implies that *THIC* may not be significantly upregulated if the HMP-PP pool is already sufficient.

It is interesting to note that 7 and 15 days post-inoculation, the expression of thiamine biosynthetic genes was lower in inoculated seedlings than in control seedlings. This suggests a tight regulatory process, whereby the genes are switched off when the thiamine pool becomes sufficient. Moreover, it indicates that the endophytic fungus *H. toruloidea* also synthesizes thiamine and therefore the thiamine biosynthetic machinery in the oil palm is repressed. Although plants generally synthesize thiamine, it may be more advantageous for them to obtain it from various external sources through biotic interactions (Helliwell et al., 2014). This was further supported by a study by Paerl et al. (2015), in which it was reported that during the co-culturing of the auxotrophic picoeukaryotic algae *Ostreococcus lucimarinus* and the bacterium *Pseudoalteromonas* sp., the algae was able to salvage thiamine from the bacterium.

In the present study, further evaluation of the effects of *H. toruloidea* colonization was performed through the quantification of total thiamine and its ester phosphates in oil palm leaves. Endophytes are known to be beneficial to the host through the production of secondary metabolites that can improve plant fitness. In this study, we assessed how colonization by the endophytic *H. toruloidea* affects total thiamine accumulation in oil palm. The significant twofold increase in the total thiamine content on day 7 post-colonization may lead to enhancement of the plant’s metabolic fitness through the activation of TPP-dependent enzymes. However, we observed that the total thiamine content was restored to control levels after 14 days, which may signal adaptive processes. Constitutive thiamine accumulation is suggested to be detrimental to the plant itself. This is because increased activity of TPP-dependent enzymes will result in the over-influx of carbohydrate oxidation through the tricarboxylic acid cycle and pentose phosphate pathway. Previous observations have shown that elevated thiamine accumulation in *Oryza sativa* overexpressing NB-LRR genes, which function as intracellular immune receptors, resulted in growth retardation and chlorosis (Wang et al., 2016). Therefore, thiamine accumulation is believed to be beneficial but only to some extent. A certain physiological level of thiamine must be maintained for optimal growth and function in plants, and this has not yet been understood.

## Conclusion

The major findings presented here demonstrate that successful colonization of oil palm seedlings by *H. toruloidea* results in the upregulation of thiamine biosynthetic genes and increased accumulation of total thiamine. Subsequent attenuation of thiamine biosynthesis signals adaptation, which may be important in maintaining optimal growth and function in plants. Further molecular, biochemical, and physiological studies are needed to understand the role and function of thiamine in the oil palm stress response.

## Author Contributions

AK performed the experiments, analyzed the data, and wrote the manuscript. ZBY designed the research, ZBY, AI, KL, and DL supervised the work and revised the manuscript.

## Conflict of Interest Statement

The authors declare that the research was conducted in the absence of any commercial or financial relationships that could be construed as a potential conflict of interest.
