# Supplementary material for: Enhancement of Thiamine Biosynthesis in Oil Palm Seedlings by Colonization of Endophytic Fungus Hendersonia toruloidea
Source: Front Plant Sci. 2017 Oct 17;8:1799. doi: 10.3389/fpls.2017.01799 (PMC5651052; doi:10.3389/fpls.2017.01799)
Supplement: Supplementary file 1 [file Table_1.DOCX]

**Supplementary materials**

Table S1. **List of primers**

| **Gene of interest** | **Forward primer (5ʹ to 3ʹ)** | **Reverse primer (5ʹ to 3ʹ)** | **Amplicon size** |
| --- | --- | --- | --- |
| **Tubulin**  **F3 R3** | ACACGGCATAGATCCAACCG | TGG TTC CAG GCT CCA AATC | 147 bp |
| **GADPH F1 R1** | GTCCCACCTGCTCAAGTACG | CGGACACGACCTTGATGACC | 110 bp |
| **THI4**  **F3 R3** | ATGATCACCCACGCCGACAC | TGCTCCACGATGGCGACTTG | 104 bp |
| **THIC**  **F3 R3** | AATGAAGGTCCAGGGCAT | GCTGAGGTGATGTGATCA | 188 bp |
| **TH1**  **F5 R5** | CCGACATGCCAGCACGAGTT | ACACCACCACAGCCAATGTAGT | 130 bp |
| **TPK**  **F5 R5** | GGGAGCACTTGGTGGAAGGTTT | TGTGGATCTCATGGCGATGTGT | 140 bp |
